# A Prospective Observational Study Evaluating the Efficacy of Omalizumab and Mepolizumab in Patients With Severe Asthma

**DOI:** 10.7759/cureus.97051

**Published:** 2025-11-17

**Authors:** Mohamed Makhlooq, Ali Qader, Mohamed Redha Salman, Zahra Al-Omran, Ameera Allawi, Thuraya Zaid, Muneer Mahdi

**Affiliations:** 1 Pulmonary Medicine, Salmaniya Medical Complex, Al-Manama, BHR; 2 Internal Medicine, Arabian Gulf University, Al-Manama, BHR; 3 Internal Medicine, Salmaniya Medical Complex, Al-Manama, BHR; 4 Medicine, Salmaniya Medical Complex, Al-Manama, BHR

**Keywords:** asthma, biologic agents, biologic therapies, refractory asthma, severe-asthma

## Abstract

Background: Severe asthma affects 5-10% of asthmatics and presents a significant challenge for healthcare systems despite therapeutic advances. Biologic therapies such as omalizumab and mepolizumab have emerged as promising treatments, but real-world comparative data remain limited.

Objective: To evaluate and compare the efficacy and safety of omalizumab and mepolizumab in patients with severe asthma in a real-world clinical setting.

Methods: This prospective observational cohort study included 72 patients with severe asthma (40 receiving omalizumab and 32 receiving mepolizumab) at the respiratory clinic of Salmaniya Medical Complex, Bahrain. The primary outcome was asthma control as assessed by the Asthma Control Test (ACT). Secondary outcomes included lung function parameters (forced expiratory volume (FEV)1% predicted and FEV1/forced vital capacity (FVC) ratio), exacerbation rates, hospitalization rates, emergency room visits, quality of life, and safety (adverse events). Data regarding demographics, asthma history, treatment details, and patient-reported outcomes were collected over a 12-month follow-up period and analyzed.

Results: The study revealed that 69.4% of all patients achieved well-controlled asthma, with the omalizumab group showing a significantly higher proportion of well-controlled patients (80.0%) compared to the mepolizumab group (56.3%, p=0.049). However, logistic regression analysis demonstrated that mepolizumab was associated with significantly higher odds of achieving high asthma control (adjusted OR: 8.95, 95% CI: 1.79-44.51, p=0.007) when controlling for other factors. Triple therapy (adjusted OR: 32.5, 95% CI: 12.85-53.11, p=0.030) and inhaled corticosteroid and long-acting beta-agonist (ICS-LABA) combination (adjusted OR: 17.43, 95% CI: 1.58-35.11, p=0.048) were strongly associated with improved asthma control. Quality of life was reported as excellent by 59.7% of patients, with a higher proportion in the omalizumab group (70.0%) compared to the mepolizumab group (46.9%).

Conclusion: Both omalizumab and mepolizumab demonstrated effectiveness in managing severe asthma, with each showing distinct advantages in different outcome measures. Mepolizumab showed stronger independent associations with asthma control in multivariate analysis, while omalizumab-treated patients reported better quality of life. Treatment selection should be tailored to individual patient characteristics and therapeutic goals.

## Introduction

Severe asthma affects approximately 5-10% of individuals with asthma and represents a disproportionate burden on healthcare resources despite comprising a minority of asthma cases [1]. These patients experience significant limitations in quality of life, frequent exacerbations, and high healthcare utilization despite receiving optimal conventional therapies [2]. The introduction of biologic therapies targeting specific inflammatory pathways has revolutionized the management of severe asthma, offering new options for patients who remain uncontrolled on standard treatments [3].

Among these biologics, omalizumab, a monoclonal antibody that targets free serum immunoglobulin E (IgE), was the first to receive approval for the treatment of severe allergic asthma [4]. Mepolizumab, which recognizes and blocks interleukin-5 (IL-5), has shown efficacy in patients with severe eosinophilic asthma by reducing eosinophil-driven inflammation [5]. Both therapies have demonstrated efficacy in reducing exacerbation rates and improving lung function in randomized controlled trials [5,6]. However, comparative real-world data on their efficacy and safety profiles are limited, particularly in Middle Eastern populations.

The relatively high cost of these biologic therapies and the heterogeneity of severe asthma necessitate a better understanding of their comparative effectiveness in real-world settings. This is particularly important for optimizing treatment selection and resource allocation in healthcare systems. Furthermore, there remains uncertainty about which patient subgroups derive the greatest benefit from each specific biologic agent [7]. This study aimed to address these knowledge gaps by evaluating and comparing the efficacy and safety of omalizumab and mepolizumab in patients with severe asthma in a real-world clinical setting at the respiratory clinic of Salmaniya Medical Complex, the largest tertiary care hospital in the Kingdom of Bahrain.

## Materials and methods

Study design and setting

This was a prospective, observational cohort study involving 72 patients diagnosed with severe asthma who were administered either omalizumab or mepolizumab. The study was conducted at the respiratory clinic of Salmaniya Medical Complex in the Kingdom of Bahrain after obtaining approval from the Research Committee for Government Hospitals (approval no. 63-230524). Both medications were already approved by the National Health Regulatory Authority (NHRA) and were used in routine treatment for severe asthma.

Study population

Eligible participants were adults (aged 18 years and above) with a confirmed diagnosis of severe asthma, prescribed either omalizumab or mepolizumab based on clinical decisions. Participants were not randomized into intervention or control groups, as the comparative groups already existed due to clinical decisions to use either of the two drugs. Informed consent was obtained from all study participants.

Data collection

Data regarding participants' demographics, asthma history, treatment details, lung function tests (PFTs), exacerbation rates, adverse events, and patient-reported outcomes (asthma control test (ACT) score and quality of life assessment) were collected. The PFTs were part of the routine follow-up care for these patients and were not performed additionally for the purpose of this study.

Outcome measures

The primary outcome was asthma control as assessed by the ACT, categorized as well-controlled (ACT ≥20), not well-controlled (ACT 16-19), or very poorly controlled (ACT ≤15). Secondary outcomes included: (a) lung function parameters: forced expiratory volume (FEV)1% predicted and FEV1/forced vital capacity (FVC) ratio measured at baseline and 12 months, (b) exacerbation rates requiring oral corticosteroids or hospitalization over 12 months, (c) hospitalization rates and emergency room visits over 12 months, (d) quality of life assessment using a standardized questionnaire, (e) safety outcomes: adverse events, serious adverse events, and treatment discontinuations. Outcomes were assessed at baseline and at 12 months of treatment. All analyses compared patients at the 12-month timepoint.

Statistical analysis

Data analysis was conducted using SPSS software, version 27 (IBM Corp., Armonk, NY). Descriptive statistics were used to summarize the bio-demographic and clinical characteristics of the study participants. Categorical variables were presented in terms of frequencies and proportions, while continuous variables were described using means and standard deviations (SD). For examining relationships between categorical variables, Pearson's Chi-square test was used, and when assumptions for the Chi-square test were not met, the Exact Probability Test was applied. For continuous variables, independent sample t-tests were used to compare means between the two treatment groups.

Additionally, binary logistic regression analysis was conducted to identify factors independently associated with high asthma control. Adjusted odds ratios (ORAs) with 95% confidence intervals (CI) were calculated to estimate the strength of associations. A p-value of less than 0.05 was considered statistically significant for all analyses. Data are presented as N (%), mean ± standard deviation (SD). All statistical tests were two‑tailed, and p < 0.05 was considered statistically significant.

## Results

Patient Demographics and Baseline Characteristics

Table 1 presents the patterns of medication use and treatment duration among the study population. The use of short-acting beta agonists (SABA) was relatively low, reported by only 11 (15.3%) patients, with similar proportions between the mepolizumab, five (15.6%), and omalizumab, six (15.0%), groups (p=0.942). Long-acting anticholinergic medications (long-acting muscarinic antagonists, LAMAs) were widely used as maintenance therapy, with 56 (77.8%) patients receiving them. The usage was slightly higher in the mepolizumab group, 26 (81.3%), compared to the omalizumab group, 30 (75.0%), though this difference was not statistically significant (p=0.526). Similarly, leukotriene modifiers were used by a large proportion of patients, 55 (76.4%), with comparable usage between the mepolizumab, 25 (78.1%), and omalizumab, 30 (75.0%), groups (p=0.756).

The use of inhaled corticosteroids with long-acting beta-agonists (ICS-LABA) was high overall, 58 (80.6%), with a slightly higher proportion in the mepolizumab group, 27 (84.4%), compared to the omalizumab group, 31 (77.5%). However, this difference was not statistically significant (p=0.464). A notable difference was observed in the use of triple therapy (inhaled ICS/LABA/LAMA), which was significantly more common in the omalizumab group, seven (17.5%), compared to the mepolizumab group, two (6.3%) (p=0.049). Methylxanthine use was rare, reported by only one patient (2.5%) in the omalizumab group and none in the mepolizumab group (p=0.368). Regarding cortisone intake, 42 (58.3%) patients reported not using cortisone, while 19 (26.4%) used it rarely, six (8.3%) occasionally, and five (6.9%) frequently. The distribution of cortisone use patterns was similar between groups (p=0.541).

**Table 1:** Medication use and treatment duration among severe asthma patients (N=72) by treatment group SABA: short-acting beta agonists. Data in N (%); χ² values and p values reported; p < 0.05 significant. *Triple therapy consists of an inhaled corticosteroid (ICS), long-acting beta-agonist (LABA), and long-acting muscarinic antagonist (LAMA).

Treatment	Total n (%)	Mepolizumab n (%)	Omalizumab n (%)	χ²	p
SABA use	11 (15.3%)	5 (15.6%)	6 (15.0%)	0.01	0.942
Anticholinergic use	56 (77.8%)	26 (81.3%)	30 (75.0%)	0.40	0.526
Leukotriene modifier	55 (76.4%)	25 (78.1%)	30 (75.0%)	0.09	0.756
ICS/LABA therapy	58 (80.6%)	27 (84.4%)	31 (77.5%)	0.54	0.464
Triple therapy	9 (12.5%)	2 (6.3%)	7 (17.5%)	3.86	0.049*
Methylxanthine use	1 (1.4%)	0 (0.0%)	1 (2.5%)	0.81	0.368
Cortisone intake				0.62	0.541
Frequently	5 (6.9%)	2 (6.3%)	3 (7.5%)		
Occasionally	6 (8.3%)	1 (3.1%)	5 (12.5%)		
Rarely	19 (26.4%)	9 (28.1%)	10 (25.0%)		
None	42 (58.3%)	20 (62.5%)	22 (55.0%)		
Treatment duration				3.11	0.212
	13 (18.1%)	8 (25.0%)	5 (12.5%)		
1–5 years	42 (58.3%)	19 (59.4%)	23 (57.5%)		
> 5 years	17 (23.6%)	5 (15.6%)	12 (30.0%)		

In terms of treatment duration, the majority of patients, 42 (58.3%), had been on treatment for one to five years, with similar proportions in the mepolizumab, 19 (59.4%), and omalizumab, 23 (57.5%), groups. A higher percentage of mepolizumab patients had been on treatment for less than a year, eight (25.0%) vs. five (12.5%) for omalizumab, while more omalizumab patients had been treated for over five years, 12 (30.0%) vs. five (15.6%) for mepolizumab. However, these differences in treatment duration were not statistically significant (p=0.212).

Lung function parameters and asthma control

Table 2 presents the lung function parameters and asthma control levels among the study population. Based on FEV1% predicted, more than half of the patients, 37 (51.4%), had severe obstruction, while 21 (29.2%) had normal lung function, nine (12.5%) had mild obstruction, and five (6.9%) had moderate obstruction. The distribution of lung function severity was very similar between the two treatment groups, with no statistically significant difference observed (p=0.992). The mean FEV1% predicted for the total sample was 83.6 ± 31.3%, with the mepolizumab group showing a lower mean (77.7 ± 29.9%) compared to the omalizumab group (89.4 ± 32.3%), though this difference was not statistically significant.

In terms of asthma control, as measured by the ACT, 50 (69.4%) of all patients had well-controlled asthma, 14 (19.4%) were not well-controlled, and eight (11.1%) had very poorly controlled asthma. The omalizumab group had a significantly higher proportion of patients with well-controlled asthma, 32 (80.0%), compared to the mepolizumab group, 18 (56.3%) (p=0.049). Conversely, the mepolizumab group had higher proportions of patients with not well-controlled, nine (28.1%) vs. five (12.5%), and very poorly controlled, five (15.6%) vs. three (7.5%), asthma compared to the omalizumab group. The mean FEV1/FVC ratio for the total sample was 79.4 ± 12.3, with the mepolizumab group having a lower mean ratio (74.1 ± 12.8) compared to the omalizumab group (82.4 ± 11.4). While this suggests better preserved lung function in the omalizumab group, the difference did not reach statistical significance (t=1.53, p=0.129).

**Table 2:** Lung function parameters and asthma control among severe asthma patients (N=72) by treatment group Values are N (%), mean ± SD; test statistic and p values shown; p < 0.05 is significant. FEV: Forced expiratory volume, FVC: Forced vital capacity.

Parameter	Total	Mepolizumab	Omalizumab	Test statistics	p-value
FEV1% predicted				χ² = 0.01	0.992
Normal function	21 (29.2%)	9 (28.1%)	12 (30.0%)		
Mild obstruction	9 (12.5%)	4 (12.5%)	5 (12.5%)		
Moderate obstruction	5 (6.9%)	2 (6.3%)	3 (7.5%)		
Severe obstruction	37 (51.4%)	17 (53.1%)	20 (50.0%)		
Mean ± SD (%)	83.6 ± 31.3	77.7 ± 29.9	89.4 ± 32.3	t = −1.39	0.168
Asthma Control Test (ACT)				χ² = 3.88	0.049*
Well‑controlled	50 (69.4%)	18 (56.3%)	32 (80.0%)		
Not well‑controlled	14 (19.4%)	9 (28.1%)	5 (12.5%)		
Very poorly controlled	8 (11.1%)	5 (15.6%)	3 (7.5%)		
FEV1/FVC ratio	79.4 ± 12.3	74.1 ± 12.8	82.4 ± 11.4	t = −1.53	0.129

Quality of life

**Figure 1:**
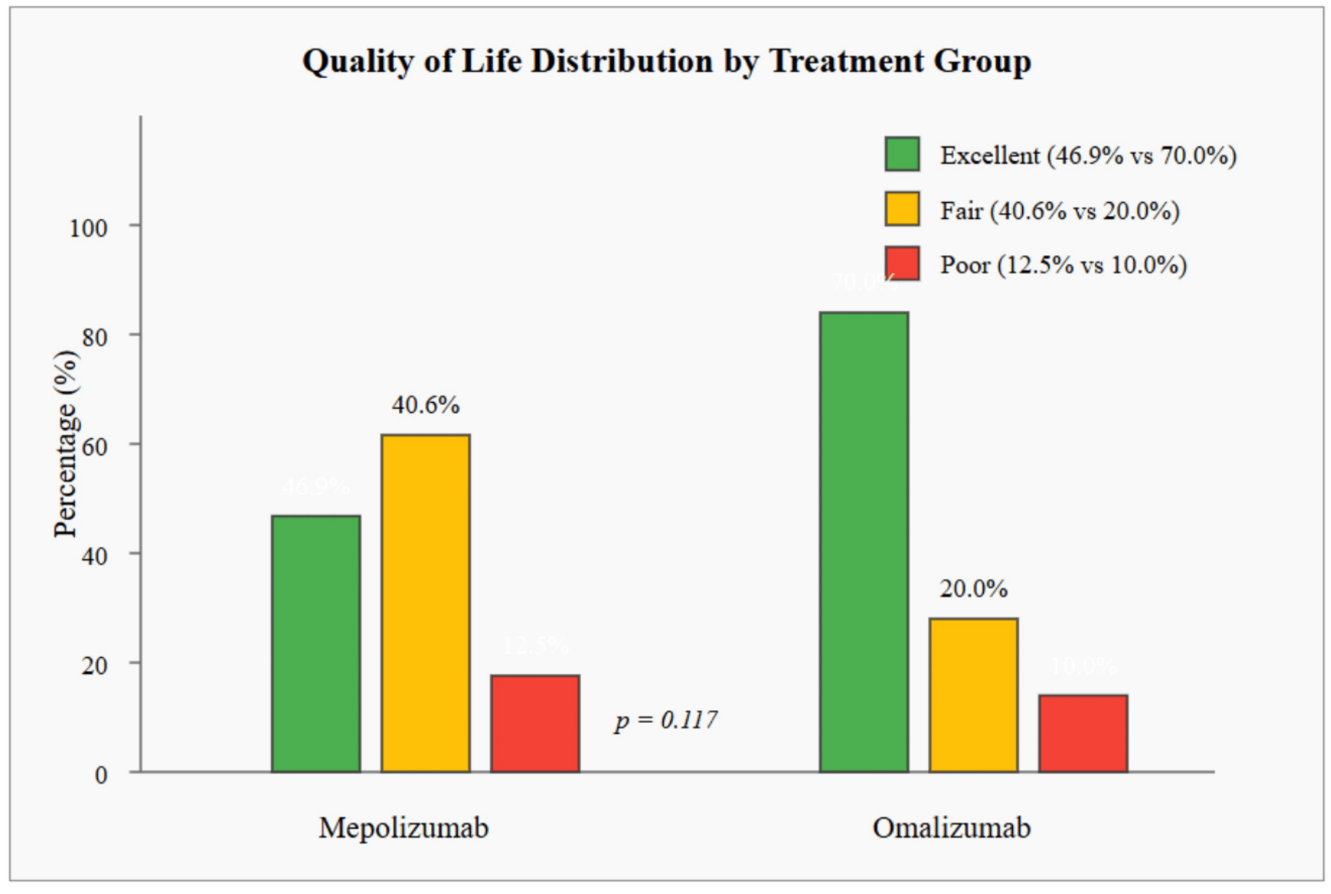
Quality of life (QoL) distribution among patients with severe asthma treated with mepolizumab and omalizumab (N=72) Data shown as percentages; statistical comparison using Pearson’s χ² (χ² = 2.45, p = 0.117). p < 0.05 considered significant.

Figure 1 illustrates the distribution of quality of life (QoL) among severe asthma patients receiving either mepolizumab or omalizumab. Overall, the majority of patients, 43 (59.7%), reported an excellent quality of life, with a higher proportion in the omalizumab group, 28 (70.0%), compared to the mepolizumab group, 15 (46.9%). In contrast, 13 (40.6%) patients in the mepolizumab group reported a fair quality of life, which was twice the proportion observed in the omalizumab group, eight (20.0%). The proportion of patients reporting a poor quality of life was similar in both groups, at four (12.5%) for mepolizumab and four (10.0%) for omalizumab, totaling eight (11.1%) across the entire sample. However, these differences in quality of life distribution between the two treatment groups did not reach statistical significance (p=0.117).

Factors associated with high asthma control

Table 3 presents the results of the binary logistic regression analysis, which identified factors independently associated with high asthma control among patients with severe asthma. The analysis revealed several significant factors. Interestingly, patients receiving mepolizumab had significantly higher odds of achieving high asthma control compared to those on omalizumab (adjusted OR: 8.95, 95% CI: 1.79-44.51, p=0.007). This suggests that when controlling for other factors, mepolizumab treatment was associated with a nearly nine-fold increase in the likelihood of better asthma control.

Shorter asthma duration was also significantly associated with better asthma control (adjusted OR: 0.94, 95% CI: 0.87-1.00, p=0.049), indicating that each additional year of asthma duration slightly reduced the odds of achieving high control. Use of ICS-LABA combination therapy was significantly associated with improved asthma control (adjusted OR: 17.43, 95% CI: 1.58-35.11, p=0.048), suggesting that patients on this treatment were much more likely to achieve control. Furthermore, those on triple therapy had the highest association with high asthma control (adjusted OR: 32.5, 95% CI: 12.85-53.11, p=0.030), indicating a very strong relationship between this intensive treatment and asthma control. Frequent corticosteroid (CS) intake was also significantly linked with high control (adjusted OR: 3.83, 95% CI: 1.16-9.10, p=0.002). In contrast, factors such as age, gender, BMI, family history of asthma, co-morbidities, duration of treatment, and use of medications like SABA, anticholinergics, and leukotriene modifiers did not show a statistically significant association with asthma control.

**Table 3:** Binary logistic regression analysis of factors associated with high asthma control (N=72) Data expressed as adjusted odds ratio (ORₐ) (95% CI); Wald χ² statistics and p values shown; p < 0.05 significant.

Factor	Wald χ²	p	ORₐ	95% CI (Lower, Upper)
Mepolizumab vs Omalizumab	7.24	0.007*	8.95	1.79 – 44.51
Age (years)	0.02	0.893	1.00	0.95 – 1.07
Female gender	0.37	0.544	1.61	0.35 – 7.49
BMI	1.00	0.317	1.04	0.96 – 1.13
FH of asthma	0.63	0.430	1.89	0.83 – 9.20
Other comorbidities	0.35	0.555	0.64	0.14 – 2.83
Duration of asthma (yrs)	3.84	0.049*	0.94	0.87 – 1.00
Duration of treatment (yrs)	0.08	0.773	1.03	0.84 – 1.27
SABA use	1.09	0.297	2.73	0.41 – 18.11
Anticholinergic use	0.81	0.369	3.50	0.23 – 53.86
Leukotriene modifier use	2.02	0.157	3.23	0.64 – 16.34
ICS/LABA therapy	3.93	0.048*	17.43	1.58 – 35.11
Triple therapy	4.72	0.030*	32.50	12.85 – 53.11
Frequent CS intake	9.33	0.002*	3.83	1.16 – 9.10

## Discussion

This prospective observational study provides valuable real-world evidence on the comparative efficacy and safety of omalizumab and mepolizumab in patients with severe asthma in Bahrain. The findings highlight important similarities and differences between these two biologic therapies in terms of their effects on asthma control, lung function, and quality of life. Our study population's demographic characteristics align with the typical profile of severe asthma patients reported in the literature, with a high prevalence of obesity, 43 (59.8%), and a substantial proportion having long-standing asthma [8]. The significant association between family history of asthma and omalizumab treatment (p=0.043) may reflect the allergic phenotype for which omalizumab is typically prescribed, as it targets immunoglobulin (Ig)E-mediated allergic responses [9].

Regarding asthma control, an interesting discrepancy emerged between the initial bivariate and subsequent multivariate analysis. In the bivariate analysis, omalizumab appeared to achieve better asthma control, with 32 (80.0%) patients having well-controlled asthma compared to 18 (56.3%) in the mepolizumab group (p=0.049). However, when controlling for other factors in the logistic regression model, mepolizumab treatment was associated with significantly higher odds of achieving high asthma control (adjusted OR: 8.95, 95% CI: 1.79-44.51, p=0.007). This apparent contradiction may be explained by the influence of confounding variables and the complex interplay of factors affecting asthma control [10].

The stronger association of mepolizumab with high asthma control in the multivariate analysis aligns with findings from previous studies demonstrating its effectiveness in reducing eosinophilic inflammation and improving control in severe asthma [5,11]. The DREAM, MENSA, and SIRIUS trials have all demonstrated significant reductions in exacerbation rates and improvements in quality of life with mepolizumab treatment [5,11]. Our analysis also identified several other factors significantly associated with high asthma control. The inverse relationship between asthma duration and control (p=0.049) suggests that longer disease duration may be associated with more refractory disease or greater airway remodeling, making control more challenging to achieve [12]. The strong associations of ICS-LABA (p=0.048) and triple therapy (p=0.030) with high asthma control underscore the importance of optimizing background controller medications even when using biologics [13].

Interestingly, despite the better asthma control associated with mepolizumab in the multivariate analysis, patients in the omalizumab group reported better quality of life. Although not reaching statistical significance (p=0.117), 28 (70.0%) of omalizumab patients reported excellent quality of life compared to 15 (46.9%) in the mepolizumab group. This discrepancy between control and quality of life highlights the complex and multifaceted nature of the patient experience in severe asthma and suggests that factors beyond symptom control contribute to patient-reported quality of life [14].

Regarding lung function, no significant differences were observed between the two treatment groups in terms of FEV1% predicted or FEV1/FVC ratio. This is consistent with findings from clinical trials showing modest effects of biologics on lung function compared to their more pronounced effects on exacerbation rates and symptom control [6,11]. The similar exacerbation rates, hospitalizations, and emergency room visits between the two treatment groups suggest that both biologics are effective in reducing the burden of severe asthma events. The mean number of exacerbations (3.7 ± 5.6 overall) remains high, indicating the challenging nature of managing this severe asthma population despite biologic therapy.

Our finding that triple therapy was significantly more common in the omalizumab group, seven (17.5%) vs. two (6.3%), p=0.049, may reflect differences in prescribing patterns or patient characteristics. Triple therapy, consisting of ICS, LABA, and a long-acting muscarinic antagonist (LAMA), is often reserved for patients with more severe or difficult-to-control asthma, and its higher use in the omalizumab group might indicate more challenging disease in these patients [15]. The relatively lower penetration of ICS-LABA combination therapy (80.6%) and particularly triple therapy (12.5%) in our cohort prior to biologic initiation warrants discussion. While current guidelines recommend optimizing these controller medications before initiating biologics, real-world practice patterns may vary due to several factors, including patient adherence challenges, insurance coverage limitations, side effects, or rapid disease progression necessitating earlier biologic intervention. This observation highlights the heterogeneity of severe asthma management pathways and underscores the importance of individualizing treatment approaches based on patient-specific factors and local healthcare contexts.

Strengths and limitations

This study has several strengths, including its prospective design, real-world setting, and comprehensive assessment of multiple outcome measures. However, it also has limitations that should be acknowledged. The relatively small sample size may have limited the statistical power to detect some differences between the treatment groups. Additionally, the study was conducted at a single center, which may limit the generalizability of the findings to other settings or populations.

## Conclusions

In conclusion, this prospective observational study provides valuable insights into the comparative effectiveness of omalizumab and mepolizumab in patients with severe asthma in a real-world setting. Both biologics demonstrated effectiveness in managing severe asthma, with mepolizumab showing stronger independent associations with asthma control in multivariate analysis, while omalizumab-treated patients reported better quality of life.

The findings highlight the importance of considering multiple factors when selecting biologic therapy for patients with severe asthma, including specific disease phenotype, comorbidities, and patient preferences. The significant associations of ICS-LABA and triple therapy with high asthma control underscore the continued importance of optimizing background controller medications even when using biologics. Future research should focus on identifying patient characteristics that predict response to specific biologics, as well as investigating the long-term outcomes and safety profiles of these treatments in diverse real-world populations. Such evidence would further refine our approach to personalized medicine in severe asthma management.

## Appendices

**Table 4:** Appendix I (case record form)

A Prospective Observational Study Evaluating the Efficacy and Safety of Omalizumab, Mepolizumab and Dupilumab in Patients with Severe Asthma		
			Participant Information	Name	___________________________________________
	Participant ID	___________________________________________
	Home Location	___________________________________________
	Date of Birth (DD/MM/YYYY)	__ / __ / ____
	Age	____
	Gender	□ Male □ Female □ Other: _____________
	Job	___________________________________________
	Weight (kg)	____
	Height (cm)	____
	Education Level	___________________________________________
	Family History of Asthma	□ Yes □ No
	Pets or Birds at Home	□ Yes □ No
	Smoking	□ Yes □ No
	If yes, specify amount	___________________________________________
	Duration	___________________________________________
Clinical Information	Asthma Step	____
	Duration of Asthma	_____ years _____ months
	Duration of Treatment	_____ years _____ months
	Asthma Quality of Life Questionnaire (AQLQ) Score	____
	Diagnosis Date of Severe Asthma (DD/MM/YYYY)	__ / __ / ____
Current Asthma Medications	Drug name	______________________________
	Dose	______________________________
	Frequency	______________________________
	Start Date	______________________________
Asthma History	Number of Exacerbations (Past 12 Months)	______________________________
	Hospitalizations (Past 12 Months)	______________________________
	ER Visits (Past 12 Months)	______________________________
Lung Function Tests	Baseline FEV1 (L)	_______ ; Date: _______
	FEV1 (L) subsequent readings	Date(s): _________________________
Treatment Details	Eosinophils (count/%)	______________________________
	IgE Level (IU/mL)	______________________________
	Inhalation Allergy (specify allergens)	______________________________
	Use of Steroids	□ Never □ Rarely □ Occasionally □ Frequently □ Depending
Adverse Events	Experienced any adverse events?	□ Yes □ No
	If yes, list and provide dates	______________________________
Patient-Reported Outcomes	Asthma Control Test (ACT) Score	______________________________
	Quality of Life Improved Since Treatment?	□ Yes □ No
	If yes, rate improvement (0–10)	0 1 2 3 4 5 6 7 8 9 10

**Table 5:** Appendix 2 (informed consent document)

Informed consent document	
Informed Consent Document for Participation in a Research Study	
Study Title: A Prospective Observational Study Evaluating the Efficacy and Safety of Omalizumab, Mepolizumab and Dupilumab in Patients with Severe Asthma	
You are being invited to participate in a research study that aims to observe and compare the outcomes of two biologic treatments, omalizumab and dupilumab, for severe asthma. This study is being conducted at the respiratory clinic of Salmaniya Medical Complex in the Kingdom of Bahrain. If you agree to participate, we will collect data related to your demographics, asthma history, details of your treatment, lung function tests, rates of asthma exacerbations, any adverse events you experience, and your reported outcomes on quality of life and asthma control. There are no anticipated risks involved by participating in this study. The potential benefits of participating include contributing to valuable research that could improve the management of severe asthma for patients in the future. Your personal information will be kept confidential, and only the research team will have access to your data. Your participation in this study is entirely voluntary. By signing below, you confirm that you have read and understood the information provided about the study, and you agree to participate in the study under the conditions described.	
Participant's Name:	
Signature:	
Date:	
